# Advances in the clinical diagnosis and treatment of multiple-level noncontiguous spinal fractures

**DOI:** 10.3389/fneur.2024.1469425

**Published:** 2024-11-21

**Authors:** Bangmin Luo, Huarong Chen, Mingxiang Zou, Yiguo Yan, Xueqian Ouyang, Cheng Wang

**Affiliations:** The First Affiliated Hospital, Department of Spine Surgery, Hengyang Medical School, University of South China, Hengyang, Hunan, China

**Keywords:** multiple-level noncontiguous, MNSF, spinal fractures, clinical diagnosis and treatment, neurological

## Abstract

Multiple-level noncontiguous spinal fractures (MNSF) are spinal fractures that involve at least 2 sites and are characterized by the presence of one intact vertebra or intact functional spinal unit between the fractured vertebrae. MNSF account for 2.5–19% of all spinal fractures. MNSF are easily missed or have a delayed diagnosis in clinical practice and their treatment is more complex than that for single-segment spine fractures. In this article, the authors briefly summarize the advances in the etiology and mechanisms of MNSF, the identification of their involved sites and their classification, diagnosis, treatment, and prognosis.

## 1 Introduction

Multiple-level noncontiguous spinal fractures (MNSF) are spinal fractures that involve at least 2 sites and are characterized by the presence of at least one intact vertebra or intact functional spinal unit between the fractured vertebrae (1–8). Some scholars have reported that MNSF (2, 7–11) account for 2.5–5.9% of all spinal fractures, while others (3, 12–19) have concluded that MNSF account for 12–19% of all spinal fractures. Variations in the incidence of MNSF can be partially attributed to discrepancies in imaging techniques employed during patient diagnosis. Moreover, numerous studies have determined that multiple-level spine fractures constitute 26.2 to 45% of all spinal fractures. Within this subset, MNSF represent approximately 29 to 39% of cases (5, 15, 16, 19, 20). These reported data indicate that MNSF is a relatively frequent occurrence in the context of multiple-level spine fractures. Therefore, it is imperative to consider the potential presence of MNSF when diagnosing and treating multiple-level spine fractures. The primary epidemiological determinants influencing the incidence of MNSF can be categorized as follows: demographic factors, where age exerts the most significant impact; underlying conditions, with ankylosing spondylitis being the most influential; and trauma-related causes, predominantly traffic accidents. In several studies, researchers have also reported that MNSF occur in 6–12% of children and adolescents (1, 4, 21, 22), thereby insisting that the incidence of MNSF is not affected by factors such as age; however, in contrast, relevant studies have shown that young patients (age less than 60 years) are at higher risk of developing MNSF than older patients (age ≥ 60 years) (8). Furthermore, it is noteworthy that, according to Lu and Koivikko et al., the prevalence of MNSF in patients with ankylosing spondylitis combined with spinal fractures (23, 24) is as high as 17.2–25%, possibly because of the unique biomechanics of their spine (25). In recent years, the frequent occurrence of high-energy injuries resulting from traffic accidents has led to an increased incidence of MNSF. Age, BMI, and rheumatoid arthritis have been identified as potential risk factors for spinal fractures (26–28). However, their specific association with the incidence of MNSF, this particular type of spinal fracture, warrants further investigation. MNSF is often accompanied by complications such as neurological injuries and other complex traumas (3, 7, 8, 13, 29). These complications can significantly impact patient prognosis, underscoring the need for heightened vigilance among spine surgeons. MNSF is frequently overlooked or diagnosed late due to a lack of comprehensive understanding of the at-risk population, its underlying mechanisms, and clinical presentations. The repercussions of a missed diagnosis of MNSF include delayed neurological damage. Furthermore, the treatment of MNSF is challenging, with existing protocols being subject to debate. Therefore, this review aims to offer pertinent references to aid in the clinical diagnosis and treatment of MNSF. This article is a review of current research advances in the clinical diagnosis and mechanisms of MNSF, their involved sites and their classification, diagnosis, treatment, and prognosis.

## 2 Causes and mechanisms

High energy trauma is more likely to cause multiple-level noncontiguous spinal fractures (24, 30). The causes of MNSF include road traffic accidents, accidental falls from heights, diving accidents, and heavy object injuries, with traffic accidents and falls from heights being the main causes (3, 7, 10, 11, 31). Compared with low-velocity trauma, high-velocity blunt trauma has a 60% increased risk of MNSF and a 6.7% increased risk of mortality (14). Interestingly, in traffic accidents, we generally believe that seat belts prevent most spinal injuries, but in a report by Nourbakhsh et al., seat belts may also be a factor in the occurrence of multiple-level noncontiguous spinal fractures, where the upper and lower shoulder and abdominal straps act as two fulcrums, thus causing two nonadjacent fracture dislocations (32). Furthermore, the main causes of morbidity in pediatric patients with multiple-level noncontiguous spinal fractures are sports-related injuries in addition to traffic accidents and high falls, while the most common causes of morbidity in elderly patients with MNSF are mainly osteoporotic spine fractures and accidental falls from a low height (38.6 and 28.6%, respectively) (1, 5, 8, 22, 33, 34). And, in elderly patients with osteoporotic MNSF, most do not have a significant history of spinal trauma (35).

**Figure 1:**
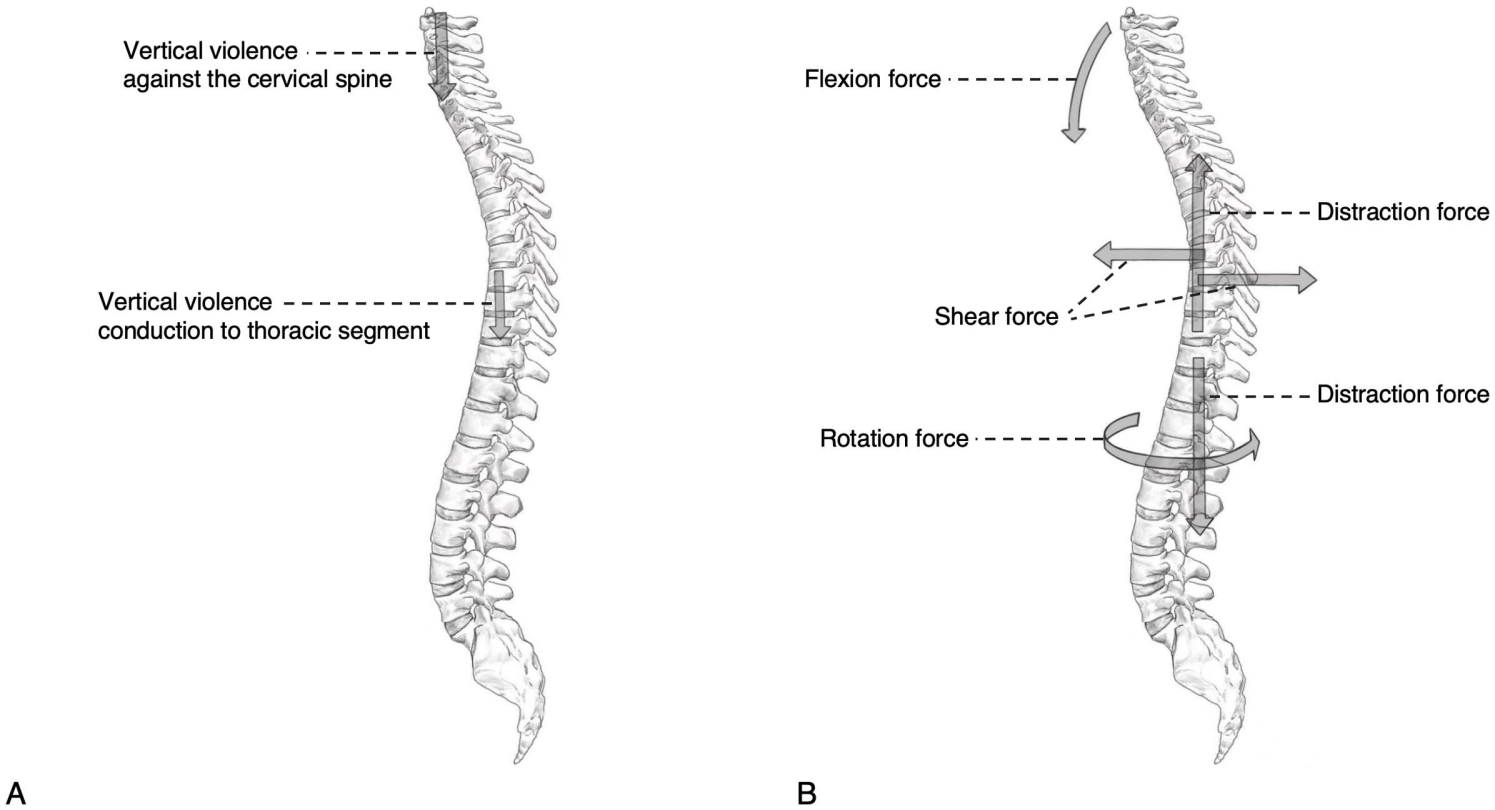
Diagram of the pathogenesis of MNSF. (A) Vertical violence conducted through the cervical spine to the thoracic spine can lead to MNSF. (B) Two or more different forces applied to different parts of the spine can lead to MNSF.

Iencean et al. (2) suggested that multiple-level noncontiguous spinal fractures may be associated with biomechanical patterns of violence conduction at the time of injury. This mechanism suggests that direct violence at the impact of a large trauma results in a first localized injury to the spine, thereby possibly causing a second injury at the vulnerable level of the spine due to the propagation of residual traumatic forces, and that spinal vulnerability may be related to the presence of spinal disease prior to spinal trauma or specific spinal body positions with discontinuous biomechanical conduction at the time of trauma (Figure 1A). In contrast, some scholars (36–38) suggest that MNSF result from the combination of excessive spinal flexion, compression, distraction and shear forces applied to the spine. This is similar to Takami et al. (7), who reported that MNSF is mainly caused by flexion, compression, distraction, and rotation forces applied to the spine, thereby resulting in injury (Figure 1B). When the spine is subjected to high-energy trauma, the spine is often subjected to several vectors of force acting together at the same time, so the exact mechanism by which MNSF occurs is complex.

## 3 Site of disease and typology

The sites of MNSF are different in different studies. Takami et al. (7) reported that 50% of MNSF occur in the cervical + thoracic region, 22.2% in the cervical + lumbar region, 11.1% in the thoracic + lumbar region, 5.6% in the cervical region, 5.6% in the thoracic region, and 5.6% in the lumbar region. While Seçer et al. (31) reported that 40% of MNSF were located in the thoracic + thoracic spine, 26.7% were located in the thoracic + lumbar spine, 20% were located in the lumbar + lumbar spine, and 13.3% were located in the cervical + lumbar spine. In addition, Korres et al. (10) showed that 28.4% of MNSF were found in the cervical spine, 24.7% in the thoracic + lumbar spine, 17.3% in the cervical + thoracic spine, 11.1% in the thoracic spine, 9.9% in the cervical + lumbar spine, 6.2% in the sacral spine, 1.2% in the thoracic + sacral spine, and 1.2% in the lumbar + sacral spine. Although the sites of MNSF are different in different studies, most of them involve the thoracic spine, reminding us of the importance of considering such fractures in this area when examining patients with spinal fractures. Notably, the unique anatomical and biomechanical factors in children, such as relative immaturity of the neck muscles, incomplete ossification of the spine, wedge-shaped vertebrae, large head proportions, and ligamentous instability, can lead to differences in fracture sites between children and adults (1, 5, 21, 30, 39–42), and the most predominant sites of MNSF in children are the cervical spine (4, 5, 39, 40). In addition, in a study on cervical MNSF by Tang et al. (19), the fractures most commonly involved C1 or C2 and C7, and their study showed that the probability of a C1 or C2 fracture in the presence of a C7 fracture was 11.9%. Regarding the classification of MNSF, in recent years, Kanna et al. (3) classified MNSF into five types based on the site of injury on the whole spine MRI scan; type I: cervical and thoracic fractures, type II: thoracolumbar and lumbosacral fractures, type III: thoracic and thoracolumbar fractures, type IV: cervical and thoracolumbar fractures, and type V: lumbosacral fractures and related injuries. This classification can effectively classify most patients with MNSF and has some clinical significance.

## 4 Diagnosis

### 4.1 Diagnostic methods and bases

**Figure 2:**
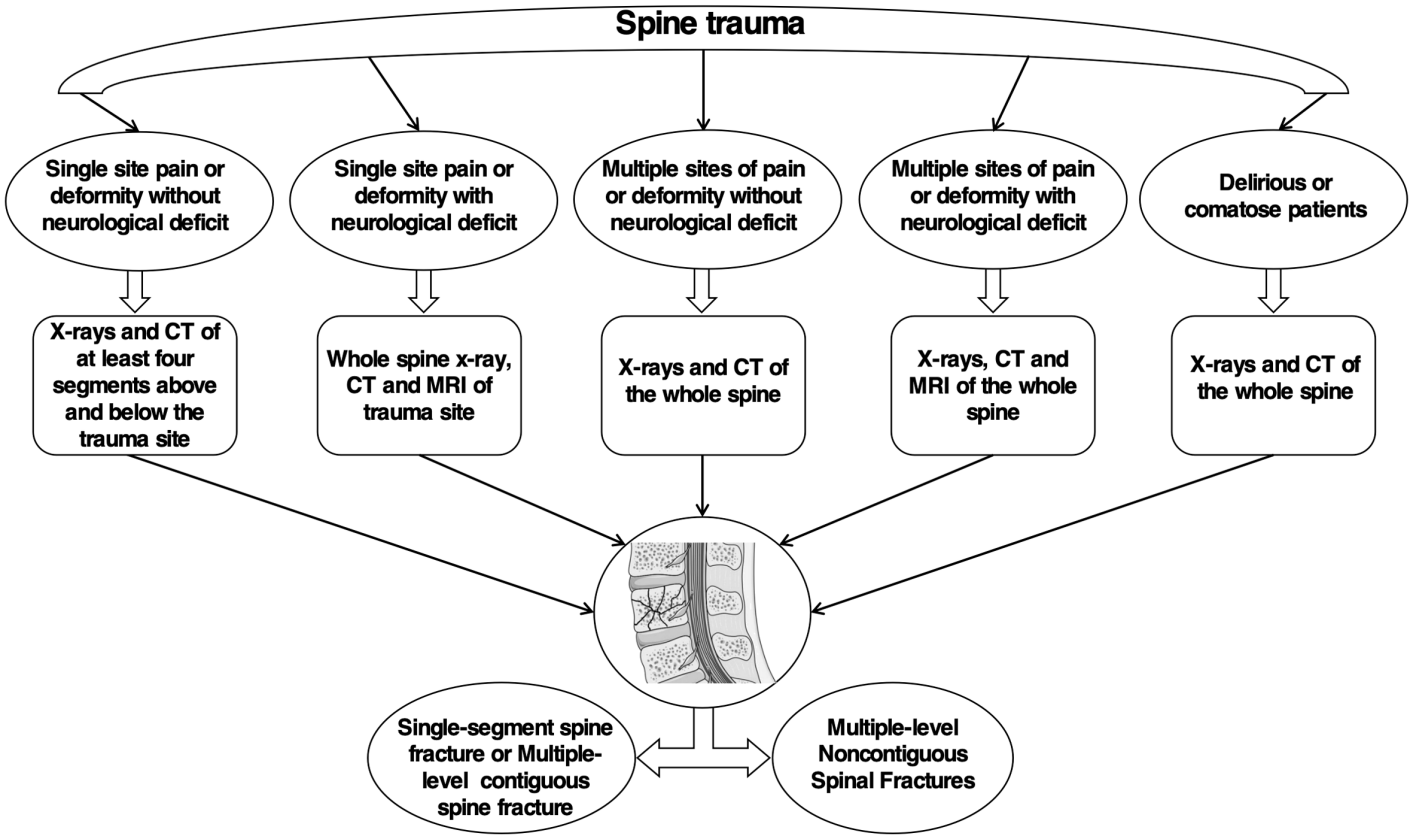
Diagnostic flow chart of MNSF. The flow chart provides a comprehensive depiction of the diagnostic process for MNSF. The flow chart is summarized based on research and our experience.

Because the incidence of MNSF in patients with spinal fractures is not insignificant, the possibility of MNSF should be considered at the first visit and the adjacent and distant segments of patients with spinal fractures resulting from high-energy trauma should be evaluated (19, 43). Additionally, a delayed or missed diagnosis is common in patients with MNSF, Firth et al. (1) reported that, in their study, 16% of second spinal fracture diagnoses were delayed in 25 patients with MNSF; Wittenberg et al. (11) reported that, in their study, 23.1% of secondary spinal lesion diagnoses were delayed in 39 patients with MNSF, and that the mean delay in diagnosis was 2.8 days, with a maximum delay of 7 days. Takami et al. (7) also reported a 14-day delay in the diagnosis of a second spinal fracture in a patient with a MNSF at the time of the primary diagnosis via X-ray. Additionally, Kanna et al. (3) reported that, among 84 patients with MNSF, 24 patients (28.6%) had a missed diagnosis of secondary fractures on X-ray, five of whom had unstable secondary injury sites that caused persistent pain, deformity, and neurological deficits and required surgical fixation. MNSF is frequently overlooked or diagnosed late due to a lack of comprehensive understanding of the susceptible population, its underlying mechanisms, and clinical manifestations. The specific mechanisms through which MNSF occurs are intricate, and the secondary injuries resulting from the violence conduction mechanism in MNSF are often neglected during the diagnostic process. Several indicators would necessitate the full spine imaging, such as abnormal findings on the physical exam (tenderness, bruises, neurological deficits), presence of a cervical spine fracture or another major distracting injury and the presence of alcohol or drug intoxication (14). Firth et al. (1) recommended that plain radiographs of the entire spine should be obtained for all patients with a single-segment spinal injury and associated nerve injury on imaging to rule out the possibility of a discontinuous injury, whereas X-ray should include at least four spinal levels above and below the fracture for patients with a single-segment spinal fracture and no nerve injury. Compared to X-rays, computerized tomography (CT) scans have a higher sensitivity in determining whether a fracture is present (44). Takami et al. (7) found that the average delay in diagnosis of the second fracture was only 0.6 days in cases where CT scan was used for diagnosis at the first visit, and therefore, their recommendation is to perform whole spine CT scans at the first visit to improve the accuracy and efficiency of a MNSF diagnosis. CT scan can provide clear anatomical images of the bony structures of the spine, facilitating the diagnosis of complex bony injuries in patients with a MNSF (45); if an unstable discontinuous spinal fracture is suspected, further evaluation of occult vertebral fractures and posterior structural damage by CT scan is necessary (46). However, excessive CT scans can somewhat increase a patient’s risk of radiation exposure. Bunmaprasert et al. (12) developed a risk prediction model for the four risk factors for MNSF that can provide guidance on whether a patient should have a CT scan. To further improve the early detection of a MNSF, magnetic resonance imaging (MRI) has also been used, and Kano et al. (34) reported that MRI is more reliable than X-ray for the diagnosis of fresh fractures in patients with a MNSF; Kanna et al. (3) suggested that whole spine MRI should be considered for patients with high energy trauma-related spinal fractures when possible to avoid a missed or delayed diagnosis. For patients with a clear location of the impact injury and significant symptoms but no abnormal signs on X-ray and CT scan, MRI has high sensitivity for diagnosing soft tissue injuries, such as bone marrow lesions, without showing evidence of a cortical fracture, thereby resulting in the exclusion of subtle nonadjacent injuries (8, 47). However, MRI can also increase the cost of patient treatment, diagnostic time, and risk of complications to some extent (44), and the decision to perform MRI should ultimately be made by the physician on a case-by-case basis (Figure 2). Meanwhile, Wang et al. (8) suggested that the cervical and thoracolumbar regions should be the primary focus of the examination in patients with MNSF because these regions are more mobile and thus more vulnerable to injury. In addition, CT scanning of the entire spine is recommended as the primary diagnostic tool for patients with ankylosing spondylitis in combination with trauma, as they are more susceptible to MNSF (48).

### 4.2 Diagnosis of comorbidities (incidence of neurological symptoms and other compound injuries)

MNSF are prone to combined neurological injury, and the incidence of combined neurological symptoms in MNSF patients is reported to be 45.5–63.2% (3, 7, 8). Firth et al. (1) showed that patients with MNSF were 2.95 times more likely to experience nerve injury than patients with single-segment or multisegment adjacent spinal injuries. The incidence of a nerve injury and its severity are closely related to the number and instability of spinal fractures (49). The presence of multiple associated injuries can interfere with the diagnosis of a secondary spinal cord injury, especially in patients with combined craniocerebral injuries, which may be missed due to an altered mental status (3). Takami et al. (7) reported that a spinal cord injury occurred in the spinal segments close to the head in eight of nine MNSF patients (Asia A, According to the American Spinal Injury Association classification) with combined complete neurological deficits in their study. The high incidence of combined nerve injury in patients with MNSF suggests the need for timely improvement of relevant investigations, with an emphasis on the proximal cephalad spinal segments and, if necessary, full-length spine films and multisite CT/MRI. For the assessment of neurological deficits in patients with MNSF, most studies currently use the American Spinal Injury Association (ASIA) score for grading. In addition, Wang et al. (8) reported a significantly different incidence of neurological deficits between young and elderly MNSF patients (57.3 and 21.4%, respectively), while injury severity scores were significantly higher in young patients (23.9 ± 10.9) than in elderly patients (15.8 ± 4.8).

Because MNSF are often caused by high-energy trauma, they are prone to concomitant nerve injuries and other compound injuries. Reports show that other compound injuries combined with MNSF mainly include head injury, neck injury, chest injury, abdominal injury, pelvic injury, extremity injury, and clavicle fracture. The most common compound injuries are chest injury, upper and lower extremity injury, and head and neck injury (3, 7, 8, 13, 29). The presence of compound injuries not only increases the difficulty and risk of treatment for patients but may also affect their prognosis and quality of life. Therefore, at the time of initial diagnosis, physicians should conduct a thorough and detailed evaluation of patients to prevent an underdiagnosis or a delayed diagnosis of associated compound injuries. Although certain patients with missed diagnoses may not necessitate immediate treatment, this situation can result in dissatisfaction and complaints from both patients and their families. Conversely, patients who require treatment but experience missed or delayed diagnoses are at risk of severe complications, including delayed nerve damage and non-healing fractures.

## 5 Treatment

### 5.1 Treatment principles

**Figure 3:**
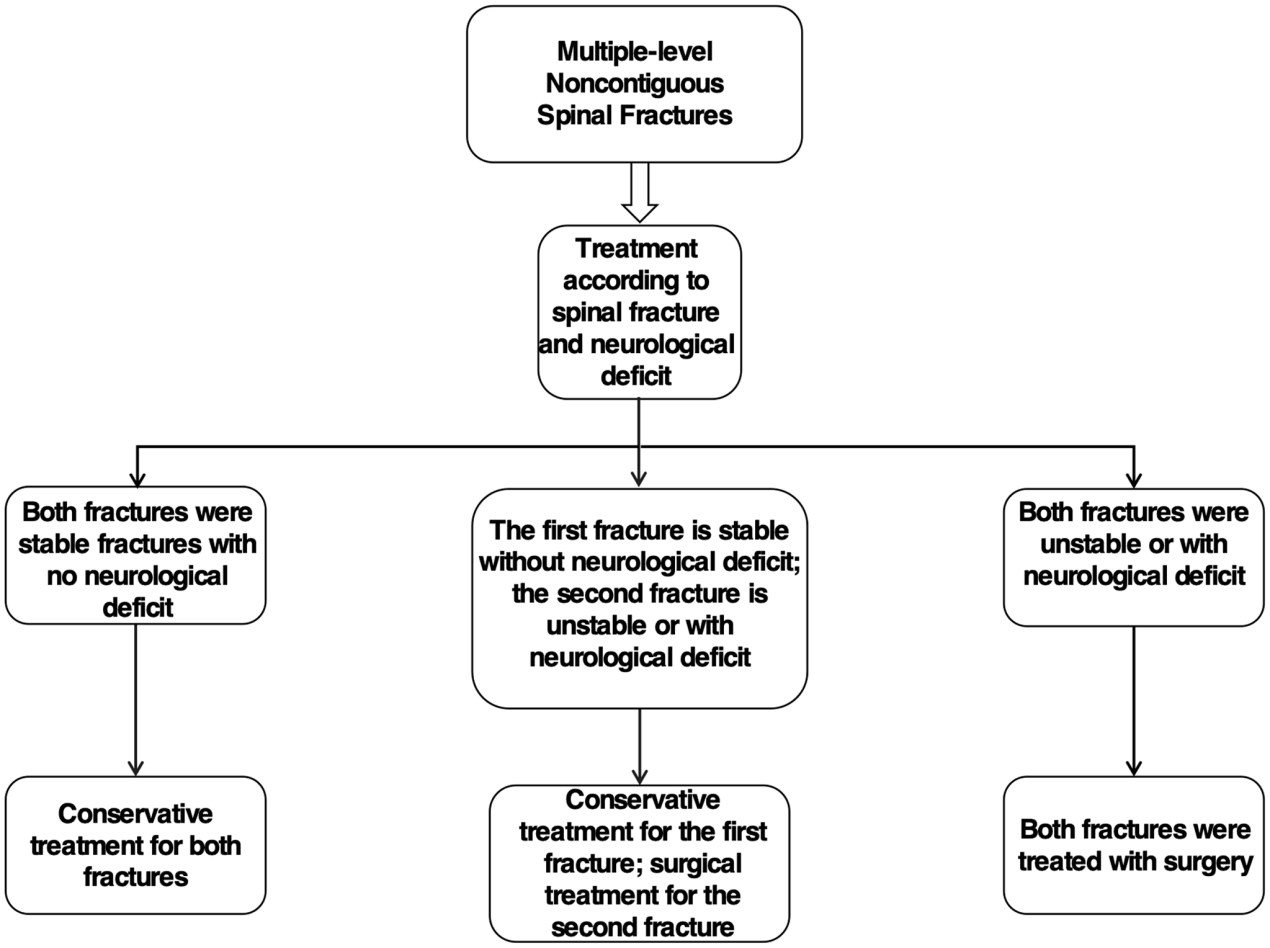
Flow chart of MNSF treatment.

The treatment of patients with MNSF should be evaluated based on the stability of the fracture and the presence of neurological impairment as well as the patient’s general condition (49). Lian et al. (29) concluded that if MNSF are stable (posterior eminence less than 30 degrees) and there is no neurological impairment, both fractures can be treated conservatively; however, if the first fracture is stable and there is no neurological impairment but the second fracture is unstable (posterior eminence greater than 30 degrees) or there is neurological impairment, only the second fracture should be treated surgically and the first fracture should be treated conservatively, whereas if both fractures are unstable (posterior eminence greater than 30 degrees) or there is neurological impairment, both fractures should be treated surgically (Figure 3). Conservative treatment of MNSF consists mainly of bed rest, Steroid therapy such as methylprednisolone, cranial traction and external brace fixation (10, 50). In contrast, surgical treatment of MNSF should follow the same principles of treatment as for single-segment spinal injuries, namely, decompression, correction of deformity, subluxation repositioning and restoration of spinal stability, with simultaneous fusion surgery of both sites if necessary (2, 5, 7, 29, 51, 52). For patients with multiple-level spinal cord injuries, early multisite decompression and internal fixation to stabilize the fracture is beneficial to improve the prognosis and reduce the risk of mortality (53). However, it is not advisable to operate on too many segments, as the preservation of segments is important to preserve the patient’s postoperative motor function (54). Cho et al. (55) reported poor KODI Disability Index scores in the long-term follow-up of MNSF patients, possibly due to too many surgically fused segments, and recommended minimizing the number of segments fused in multiple-level fractures and no reduction. In addition, the mechanism of MNSF is complex, and the management of its combined other compound injuries should follow the principles of treatment of multiple injuries, of which effective life support is a key aspect (29). However, the specific treatment plan for MNSF should be individualized according to the patient’s spinal injury site, spinal instability and deformity, severity, number of intact vertebrae between the two fractured segments, and combined neurological impairment (10, 29, 31, 56, 57).

### 5.2 Choice of surgical approach

Wang et al. (8) reported that 66.2% (141 cases) of 213 patients with MNSF underwent surgical treatment, including 47 percutaneous vertebroplasties, 2 anterior minimally invasive surgeries, 2 posterior minimally invasive surgeries, 35 anterior open surgeries, 50 posterior open surgeries, and 5 combined anterior and posterior surgeries. Based on the high proportion of patients with MNSF who underwent surgical treatment, it is particularly important to choose the appropriate surgical procedure. Several studies (2, 43, 58) have reported that anterior single-segment decompression with implant fusion of unstable lesions is mostly used for patients with MNSF with single-focal unstable fractures of the cervical spine with an intact posterior vertebral column, and they concluded that anterior cervical surgery is safer and has less bleeding than posterior surgery and provides adequate decompression of the spinal canal. However, for MNSF patients with multisite instability fractures of the cervical spine and posterior vertebral column instability, three-column injury or difficult repositioning, Jin et al. reported (59) that posterior cervical surgery or combined anterior–posterior surgery should be considered, through which posterior surgery can directly release the joint strangulation and remove the lamina, synovial fragments and ruptured ligamentum flavum that protrudes into the spinal canal. In contrast, combined anterior–posterior surgery can achieve decompression and stabilization of multisegment spinal injuries in a short period, leading to favorable conditions for neurological recovery and facilitating early functional exercise (60). For unstable fractures of the thoracolumbar spine, several investigators have used posterior open reduction fusion with pedicle screw internal fixation and vertebral body formation (7, 61–66). The anterior approach to the thoracolumbar spine is relatively complex, and posterior subtotal resection decompression fusion surgery is an effective and safe method for treating thoracolumbar fractures, resulting in less intraoperative blood loss, fewer complications, shorter operative time, less postoperative pulmonary impairment, and adequate surgical visualization than the anterior approach, and anterior internal fixation can also be replaced by posterior cage placement and bone grafting through an extended posterolateral approach (67, 68). In addition, patients with MNSF who have severe comorbidities and cannot tolerate multisite open surgery can be treated with percutaneous surgical techniques. Sebastian et al. (69) reported a case of ankylosing spondylitis combined with C1 fracture, C7-T1 three-column fracture, occipital-cervical ligament injury with dislocation, and T9-T10 three-column extension fracture in a patient who underwent open occipitocervical and cervicothoracic fusion combined with percutaneous thoracolumbar stabilization, and the patient recovered well after surgery. This hybrid surgical approach minimized the trauma caused by open surgery and created good conditions for patient recovery. At the same time, when dealing with patients with complex MNSF, we should pay attention to the combination of available tools and relevant new technologies to achieve the best treatment goals. According to Ushijima et al. (70), a patient with traumatic multiple-level noncontiguous spinal fractures at the cervicothoracic and thoracolumbar junction who was treated with a computer-assisted open posterior internal fixation hybrid technique based on a CT navigation system and percutaneous pedicle screw fixation recovered well and was able to ambulate early.

### 5.3 Selection of surgical fixation

The choice of segment length for surgical fixation in MNSF patients is clinically controversial, and although long-segment posterior pedicle screw (LSPF) fixation can give greater immediate stability at the fracture site by increasing the length of longitudinal implant fixation, it does so at the expense of the corresponding motion segment, and long-segment internal fixation exposes patients to greater stress and may expose them to a high risk of segmental degeneration adjacent to the fixation (71). In contrast, although short-segment fixation preserves more motion segments with less surgical bleeding, it also carries the risk of spinal instability due to insufficient flexion resistance. To address these issues, Seçer et al. (31) suggested that when there are ≥5 intact vertebral bodies between two fracture segments in patients with MNSF, each fracture area should be stabilized with a separate surgical incision and approach (i.e., short-segment fixation); when there are ≤4 intact segments between two fracture segments, both fractures should be fixed with the same rod and screw system (i.e., long-segment fixation). Salehani et al. (6) reported that in MNSF patients with fractures involving the anterior, middle, and posterior columns, the traditional use of a posterior approach for three vertebrae above the level of the proximal head fracture and two vertebral segments below the level of the caudal fracture with incision and pedicle screw fixation is no longer sufficient to provide good stability to the damaged spine due to the instability of the intermediate segments between the two fractures, and the use of internal fixation extension and anterior–posterior multibar structural fixation is recommended. In addition, patients should be treated with external fixation when necessary, depending on the patient’s specific situation. Wang et al. (72, 73) developed a tandem external spinal fixation for MNSF, inspired by single-segment external spinal fixation, and demonstrated by finite element analysis that the tandem external spinal fixator had better stress distribution and higher overall mobility compared with long-segment internal spinal fixation. Hope et al. (74) treated a pediatric patient with multisegmental nonadjacent spine fractures of the cervical spine who was treated with a modified external fixation frame due to the unavailability of a suitable internal fixation implant and found good neurological recovery (from ASIA grade D to ASIA grade E) at the postoperative follow-up. Meanwhile, Sane et al. (42) reported that a pediatric MNSF patient with a modified external fixation frame who underwent cranial traction repositioning using a halo ring recovered full neurological function after surgery (from ASIA class A to ASIA class E). In addition, there are studies on the use of nonfusion fixation to treat MNSF. Kim et al. (75) treated a patient with a lumbar MNSF using a minimally invasive percutaneous short-segment pedicle screw fixation nonfusion technique with spinal decompression, after which the patient’s neurological function changed from ASIA class C to normal. This technique provides some stability and facilitates the preservation of the corresponding spinal motion segments, thus providing a new option for the treatment of patients with MNSF.

## 6 Prognosis

Patients with MNSF require longer hospital stays, more days of mechanical ventilation, and longer intensive care stays than patients with single-segment spinal fractures (14). The prognosis of patients with MNSF who are surgically treated versus that of patients who are nonsurgically treated also differed significantly. Lian et al. (29) showed that the time to use a wheelchair or crutches for mobility was 9.2 ± 1.1 weeks in conservatively treated patients with MNSF, which was significantly longer than that of patients in the single lesion surgery group (6.8 ± 0.7 weeks) and that of those in the double lesion surgery group (3.1 ± 0.4 weeks), and the neurological deficit rate, improvement rate and degree of correction of the posterior protrusion deformity at the postoperative follow-up were also better in the surgically treated patients than in the conservatively treated patients, while the angle of the posterior protrusion deformity of the nonsurgical lesion of the MNSF patients increased after treatment. Wittenberg et al. (11) also showed that patients with MNSF could be more active earlier and had a reduced incidence of kyphosis after surgical treatment. In contrast, in a case of a five-segment nonadjacent spinal fracture of the cervical spine reported by Guo et al. (36), the patient refused surgical treatment due to their financial status and was discharged with unsatisfactory neurological recovery, suggesting that aggressive surgical treatment for MNSF patients with surgical indications can be helpful in improving the prognosis. This was also found to be relevant for the MNSF patients in the study by Wang et al. (8), in which 9.4% (20/213) of patients with incomplete neurological deficits improved by 1 or > 1 Asia grade during hospitalization. In addition, there are relative peculiarities in pediatric patients. Firth et al. (1) reported the presence of mild scoliosis in their follow-up of pediatric MNSF patients with combined neurological injuries. A report by Mortazavi et al. (5) also concluded that the periosteum and surrounding soft tissue enveloping the spine are more elastic in pediatric MNSF patients than in adults; therefore, bone healing and remodeling are more likely, and long-term follow-up is recommended to understand the development of a spinal deformity.

## 7 Conclusion

In summary, MNSF is a common specific type of spinal fracture with a complex pathogenesis. MNSF are easily missed and prone to delayed diagnosis, thereby requiring imaging to further clarify the diagnosis based on a comprehensive evaluation of the patient’s condition, and treatment often requires individualized treatment plans. The treatment of MNSF, especially the preferred treatment option, is still unknown, and further research by spine surgeons is needed.
